# Does neonatal ankyloglossia interfere in the growth of infants during the first 6 months of life? A case series nested in a cohort study

**DOI:** 10.1186/s13256-022-03578-2

**Published:** 2022-10-29

**Authors:** Christyann Lima Campos Batista, Alex Luiz Pozzobon Pereira

**Affiliations:** 1 Human Milk Bank, University Hospital, Federal University of Maranhão, Rua Silva Jardim, 215, São Luís, Maranhão CEP: 65020-560 Brazil; 2 Department of Dentistry, Federal University of Maranhão, São Luís, Brazil

**Keywords:** Ankyloglossia, Growth, Breastfeeding, Case report

## Abstract

**Background:**

Ankyloglossia is commonly reported as one of the major causes of breastfeeding difficulty. There is a lack of research on infant growth and latching performance with clinical measures.

**Cases presentation:**

We describe a series of eight clinical cases (three female and five male infants) in a specialized breastfeeding center in a tertiary hospital in Northeast Brazil. The mothers were of mixed race and ranged from 13 to 41 years of age. Ankyloglossia was diagnosed within the first 48 hours after delivery. We measured the standards of growth, the mothers’ perception of breastfeeding, and a pain indicator, and performed an assessment of breastfeeding. The regularity of breastfeeding was maintained despite the early diagnosis of ankyloglossia. Growth indicators were not affected in the sixth month in any of the babies, with only one measuring below expectations in the third month, with no impact on general health.

**Conclusions:**

In the cases reported in this paper, the infants overcame the initial difficulties in breastfeeding and maintained their normal growth course in the first 6 months of life.

## Introduction

Ankyloglossia has been recognized as one of the causes of breastfeeding issues due to the baby’s inability to perform or sustain latching, which can result in breast pain, nipple trauma, and early weaning [1]. The scientific community frequently reiterates the benefits of exclusive breastfeeding (EBF) until the sixth month of life, highlighting the importance of preventing illnesses and morbidities during this time [2]. Healthcare providers who interact with the mother–infant dyad must be prepared to intervene in the most common lactation issues that may impede breastfeeding [3]. The Brazilian Ministry of Health (MH) adopted legislation that requires all maternity institutions in the country to adhere to a treatment regimen that ensures proper diagnosis and follow-up of babies with ankyloglossia (Federal Law 13.002/2014). The MH protocol recommends management during the perinatal hospitalization to avoid proximal outcomes associated with early weaning and distal outcomes on the prevention of tongue functional problems such as deglutition disorders, phonetics articulation, and malocclusions such as open bite, abnormal separation of incisors, and other mechanical problems associated with oral cleaning, as well as psychological distress [4, 5].

Ankyloglossia, whether symptomatic or not, has been related to early issues in breastfeeding (BF), including poor attachment and nipple trauma [6]. However, investigations are usually inconclusive owing to diagnostic difficulties or limitations in research design [7]. The symptomatic diagnosis of ankyloglossia can be skewed as the development of stomatognathic functions may result in an adaptive stage of the tongue, capable of performing all its functions without the need for immediate intervention [8]. Although frenotomy is a straightforward and effective procedure for the vast majority of symptomatic patients, it has risks such as infection, minor bleeding, pain and discomfort, and salivary duct damage [5].

The purpose of this study was to examine the development of growth in infants with ankyloglossia throughout the first 6 months of life.

## Cases presentation

We followed up on eight patients diagnosed with ankyloglossia who was born at the Maternal-Infant Unit of the University Hospital of the Federal University of Maranhão (HUUFMA), in São Luís. The projected population is 1,101,884, with a minimum wage of 1212,00 Brazilian Reais (R$). The HUUFMA is a state-recognized tertiary hospital specializing in maternity and child health care. The infants were assessed 48 hours after delivery by two speech therapists.

The Ethics Committee for Human Research gave its approval to this study (no. 3.052.208). All infants’ parents were informed about the study’s aims and protocols, and they were encouraged to sign the informed consent form to participate. This is the preliminary outcome of a large cohort study being conducted at the institution.

The Bristol Tongue Assessment Tool (BTAT) [9], and the Hazelbaker Assessment Tool for Lingual Frenulum Function (HATLFF) were used to make the diagnosis [10]. To avoid bias in case selection, ankyloglossia was evaluated when the child had a different score on both instruments (BTAT ≤ 5 on a scale of 0–8; the appearance test scores range between 0 and 10, where a score is considered altered if ≤ 8, and function score ≤ 11 on a scale of 0–14 for HATLFF).

Following the first evaluation, the infants were followed up monthly until they were 6 months old. The LATCH scale (latch, audible swallowing, type of nipple, comfort, hold) was used to assess breastfeeding performance at admission and after the follow-up period [11]. Scores ≤ 8 indicate symptoms of breastfeeding problems.

The mothers’ assessment of the quality and the amount of pain felt while nursing was gathered during the first and last follow-up visits using the Breastfeeding Self-Efficacy Scale—Short Form (BSES-SF) [12] and the Short Form of the McGill Pain Questionnaire (SF-MGPQ) [13]. The higher the score, the greater the mother’s assessment of the efficacy of breastfeeding and the greater the pain or discomfort experienced when nursing.

During all consultations, data on the infants’ weight and length were obtained, and body mass index (BMI) and weight gain between consultations were computed using this information. The newborns were weighed on the “Balmak ELP25BB” scale without diapers. Length was measured using the horizontal stadiometer “Seca Mod. 416” (child lying down).

The World Health Organization software was used to compute BMI and age index *Z*-score data (WHO Anthro software v3.2.2). Weight gain was estimated by dividing the difference between the current weight and the previous weight by the number of days between visits.

Table 1 describes the basic characteristics of the patients who were followed. Male patients outnumbered female patients. The average maternal age was 28.1 years [standard deviation (SD) 7.43]. All the infants were delivered on time and without any abnormalities that may have hampered their growth.

**Table 1:** Data from the eight cases included in the follow-up study

Case	Infant sex	Mother’s age	Apgar score 1′/5′	Does the family have a historic of ankyloglossia?	Skin-to-skin contact at birth	Breastfed in the first hour	Breastfeeding early problems	Type of delivery	Anthropometric data at birth		
									Birth weight (g)	Cephalic perimeter (cm)	Height (cm)
1	Female	21	5/8	No	No	Yes	Nipple trauma	Cesarean	2700	33	47
2	Male	19	9/9	Yes	Yes	No	Nipple trauma	Vaginal	3145	34	45
3	Male	33	9/9	No	Yes	No	None	Vaginal	2545	33	48.5
4	Male	13	8/9	No	No	No	None	Vaginal	3217	36	51.1
5	Male	27	8/9	Yes	Yes	No	Nipple trauma	Vaginal	2745	32.5	47.5
6	Female	41	9/9	Yes	Yes	Yes	Nipple trauma	Vaginal	3750	35	51
7	Female	27	8/9	Yes	Yes	Yes	None	Cesarean	3240	34.5	48
8	Male	34	9/9	Yes	Yes	No	None	Cesarean	3880	37	49

Case 2 demonstrated weaning from the first appointment, introducing formulas combined with breastfeeding. In addition to mentioning pain when latching, the mothers said that the infant had grown accustomed to taking supplements and could no longer be weaned from them. Case 1 stated that they weaned the infant at 2 months since the baby was hungry and could not survive solely on EBF. Case 5 reported poor milk supply at 5 months and began supplementing. All other dyads breastfed exclusively until the sixth month.

Table 1 also reveals that half the mothers experienced early nipple fissure issues. Only three patients had no family history of ankyloglossia . Natural childbirth and skin-to-skin contact were also prevalent. Only three patients reported breastfeeding in the first hour.

Table 2 shows how the evaluation instruments has changed over time. After the sixth month, only two cases were recommended for corrective surgery (cases 2 and 8). The causes for the behavior were, respectively, the persistence of pain when nursing (albeit it was not regarded as debilitating pain) and significant restriction of lingual mobility. The BTAT findings for case 8 revealed a wide restriction of functional characteristics, which was verified by HATLFF.

**Table 2:** Values of breastfeeding self-efficacy, pain, and breastfeeding scores at start and end of follow-up

Case	BSES-SF		SF-MPQ		LATCH score		Weight in grams (weight gain in g/day)			BMI (*Z*-score for age)			Underwent Frenotomy	Situation at discharge
	1 month	6 months	1 month	6 months	1 month	6 months	1st month	3rd month	6th month	1st month	3rd month	6th month		
1	41	51	30	6	9	10	3830 (32.28)	5470 (19.37)	6612 (16.83)	13.06 (−0.86)	16 (−0.32)	17.2 (0.56)	No	No alteration
2	65	52	19	13	10	8	4248 (33.42)	6792 (41.66)	8610 (12.41)	15.7 (0.37)	18.2 (0.89)	19.3 (1.28)	Yes	Pain persistence
3	61	46	1	2	10	10	3340 (32.33)	5374 (28.44)	7438 (11.6)	13.9 (−0.43)	16.8 (0.05)	17.9 (0.36)	No	No alteration
4	61	56	3	0	9	10	3140 (−6.81)	6286 (44.37)	8462 (28.05)	11.6 (−1.52)	16.6 (−0.23)	18.5 (0.75)	No	No alteration
5	67	61	4	0	9	10	3868 (35.09)	5358 (17.57)	6522 (10.4)	14 (−0.79)	14.2 (−2.03)	14.8 (−1.97)	No	No alteration
6	63	61	19	2	10	10	4662 (26.8)	6122 (22.40)	7758 (12.0)	15.4 (0.41)	17 (0.40)	17.8 (0.56)	No	No alteration
7	51	63	6	0	10	10	4412 (37.8)	7242 (26.82)	7832 (12.27)	15.1 (0.33)	18 (0.77)	18.4 (0.94)	No	No alteration
8	70	70	13	0	10	9	5416 (49.54)	7666 (29.72)	9412 (17.02)	17.3 (1.59)	19.9 (1.94)	21.3 (2.44)	Yes	Tongue movement restrictions

Anthropometry in the first, second, and third months of life

*BSES-SF* Breastfeeding Self-Efficacy Scale—Short Form, *SF-MPQ* Short Form of the McGill Pain Questionnaire, *LATCH* latch, audible swallowing, type of nipple, comfort, hold, *BMI* body mass index

Although some cases had less weight gain for their age, no infant had a change in the BMI *Z*-score of less than one standard deviation, indicating that all cases had weight and height deemed appropriate for their age. Case 5 was the sole exception, with not only minimum weight gain but also a standard deviation of less than 1, indicating thinness. This was the only case where the patient had used a pacifier since the beginning of follow-up, and the mother had reported poor milk supply.

## Discussion

**Fig. 1:**
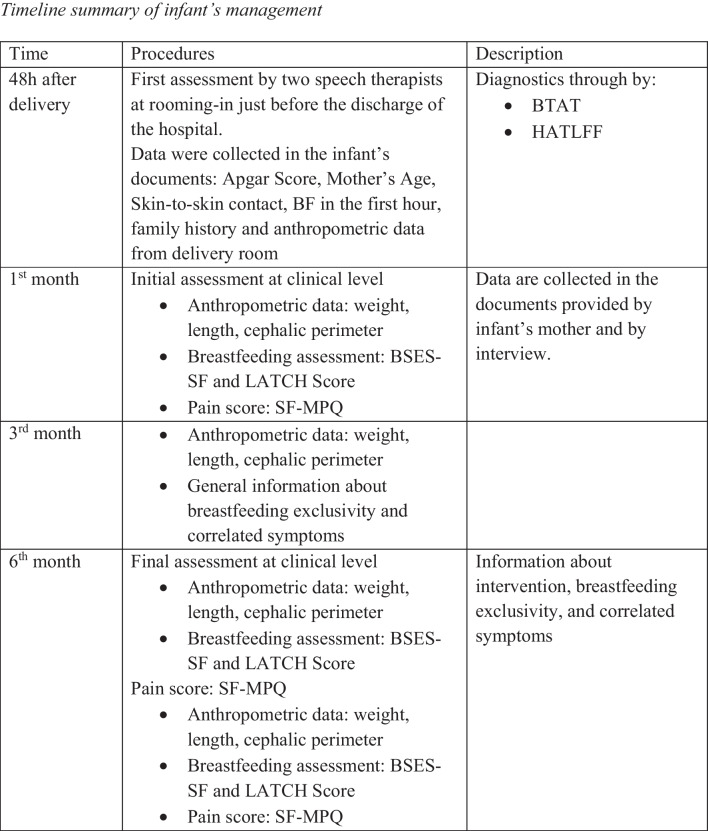
Timeline summary of infants’ management. *BF* breastfeeding, *BTAT* Bristol Tongue Assessment Tool, *HATLFF* Hazelbaker Assessment Toll for Lingual Frenulum Function, *BSES-SF* Breastfeeding Self-Efficacy Scale—Short Form, *SF-MPQ* Short Form of the McGill Pain Questionnaire, *LATCH* latch, audible swallowing, type of nipple, comfort, hold

The goal of this case study was to record the progression of the growth of newborns diagnosed with ankyloglossia, to see if, after the initial difficulties characteristic of breastfeeding, the babies would display developmental alterations or other signs of difficulties in the future (Fig. 1).

Even after a positive diagnosis was made, the majority of the patients proceeded to EBF and grew normally.

Even though the diagnosis was given in the initial days of life, the nursing scores did not indicate any problems. The link between ankyloglossia and nursing difficulties is frequently reported in the literature because the bulk of research including the diagnosis takes place in the immediate postpartum period [3, 14]. However, there are claims of insufficient funds to carry out this type of analysis [15]. The absence of experimental research, which is sometimes ethically conflicted, impedes the confirmation that ankyloglossia is the sole factor in initial difficulties observed in breastfeeding.

Observational studies have shown that tongue function is essential in developing proper suction during breastfeeding, and in this way ankyloglossia could prevent the infant from applying the proper mechanism [1, 3, 16]. Sucking is a complex process that involves the tongue and jaw in a series of compression movements and differences in pressure that allow milk transfer and adequate development of the stomatognathic system [8]. Successful breastfeeding involves a complex interplay of mother and child and correct coordination of sucking and swallowing by the infant [1].

Indicators of pain did not affect the cases studied. The case with the highest indicator on the scale exhibited normal indicators of development and breastfeeding. Pain has been identified as an essential finding in these individuals because it is a debilitating factor that might impede the formation and maintenance of EBF [17]. Ineffective sucking induced by ankyloglossia can cause discomfort and reduce weight gain, resulting in excessive breastfeeding and affecting early breastfeeding discontinuation [18]. Other variables, such as attachment and position issues, might, however, impact the initial challenges of breastfeeding [19]. In certain situations, the link between breastfeeding problems and ankyloglossia may be skewed, because most studies conduct an assessment with infants during a time when most mothers have breastfeeding issues.

Nonsurgical intervention has previously been demonstrated to be beneficial in lowering the number of procedures performed on newborns [20]. As a result, pain markers cannot be used to compile a list of the effects of ankyloglossia during the puerperal phase.

In this research, there were no cases of complete weaning, and most patients breastfed exclusively until the sixth month. Weaning is caused by a multitude of factors, ranging from societal factors to the usage of artificial teats [3].

The hospital where the monitored babies were delivered is a Baby-Friendly Hospital, an effort that has been shown to minimize weaning markers via the implementation of optimal prenatal care, delivery, and post-discharge follow-up procedures [21]. With the advocacy of exclusive breastfeeding, families are constantly reminded of the dangers of early breastfeeding interruption. Even with a positive diagnosis, no case indicated an absolute inability to justify discontinuing exclusive breastfeeding or even early intervention, which contradicts the literature [22].

Findings on the effect of changes in infant growth markers have been published, even if indirectly. A systematic review reported an increase in milk transfer and production in a group of six mothers [23]. Low weight gain has also been observed in a retrospective study of frenotomy follow-up patients [18, 24]. The continual advice provided during consultations, as well as the fact that patients are monitored in a facility specializing in breastfeeding, can help to explain the lack of substantial changes in the children’s growth.

We emphasize that establishing a cause-and-effect relationship between ankyloglossia and the outcome commonly reported in the literature is not possible; however, it is noteworthy that this series of cases can suggest information about the intimate relationship between breastfeeding difficulties and ankyloglossia, stimulating scientific debate on the subject. More research, particularly experimental research, is needed to investigate the link between the various treatment choices for infants with ankyloglossia.

There are presently just five randomized controlled trials investigating the link between ankyloglossia and breastfeeding. There were only 317 patients in the research, and the usefulness of frenotomy in enhancing the success of breastfeeding reported by mothers was verified in four of them. No study presented data on nonsurgical treatments, the subject of this case study, which revealed that breastfeeding is often unaffected even when ankyloglossia is present [7].

Extended follow-up appears to be an important component of efficient breastfeeding in babies with ankyloglossia, especially in the first months of life. In this study, patients were followed up by a multidisciplinary care team that comprised speech therapists, nurses, and a pediatrician. Patients were encouraged to continue exclusive breastfeeding for as long as possible unless they had a severe illness that justified abandoning the practice. Correct training for the teams who engage with these patients can aid in reducing diagnostic errors and, as a result, the frequency of unnecessary procedures [25].

## Conclusions

The cases described in this study show that newborns diagnosed with ankyloglossia in the immediate postpartum period can overcome the initial breastfeeding difficulties and attain normal development. This finding suggest that nonsurgical management could improve breastfeeding outcomes and reduce the number of unnecessary procedures.

## Data Availability

Not applicable.
